# Hepatitis C Virus Affects Tuberculosis-Specific T Cells in HIV-Negative Patients

**DOI:** 10.3390/v12010101

**Published:** 2020-01-15

**Authors:** Mohamed Ahmed El-Mokhtar, Sherein G. Elgendy, Abeer Sharaf Eldin, Elham Ahmed Hassan, Ali Abdel Azeem Hasan, Muhamad R. Abdel Hameed, Douaa Sayed, Eman H. Salama

**Affiliations:** 1 Department of Medical Microbiology and Immunology, Faculty of Medicine, Assiut University, Assiut 71515, Egypt; shereinelgendy@yahoo.com; 2 Department of Tropical Medicine and Gastroenterology, Faculty of Medicine, Assiut University, Assiut 71515, Egypt; sharafabeer@yahoo.com (A.S.E.); mam_elham75@yahoo.com (E.A.H.); 3 Department of Chest Diseases and Tuberculosis, Faculty of Medicine, Assiut University, Assiut 71515, Egypt; aabdelazeem@yahoo.com; 4 Internal Medicine and Hematology Unit, Assiut University Hospitals, Assiut University, Assiut 71515, Egypt; dr.muhamadramadan@yahoo.com; 5 Department of Clinical Pathology, South Egypt Cancer Institute, Assiut University, Assiut 71515, Egypt; Douaa_sayed@hotmail.com; 6 Department of Clinical Pathology, Faculty of Medicine, Sohag University, Sohag 82524, Egypt; eman_salama@med.sohag.edu.eg

**Keywords:** tuberculosis, hepatitis C virus, TB/HCV coinfection, T cells, IL-10

## Abstract

The occurrence of tuberculosis (TB) and hepatitis C virus (HCV) infections in the same patient presents a unique clinical challenge. The impact of HCV infection on the immune response to TB remains poorly investigated in TB^+^/HCV^+^ patients. This study was conducted to evaluate the impact of HCV on the T-cell-mediated immune response to TB in coinfected patients. Sixty-four patients with active TB infections were screened for coinfection with HCV. The expression of immune activation markers IFN-γ, CD38, and HLA-DR on TB-specific CD4^+^ T cells was evaluated by flow cytometry in TB-monoinfected patients, TB/HCV-coinfected patients, and healthy controls. IL-2, IL-4, IFN-γ, TNF-α, and IL-10 levels were measured using ELISA. The end-of-treatment response to anti-TB therapy was recorded for both patient groups. Significantly lower levels of CD4^+^IFN-γ^+^CD38^+^ and CD4^+^IFN-γ^+^HLA-DR^+^ T cells were detected in TB/HCV-coinfected patients compared to TB monoinfected patients and controls. TB^+^/HCV^+^-coinfected patients showed higher serum levels of IL-10. The baseline frequencies of TB-specific activated T-cell subsets did not predict the response to antituberculous therapy in TB^+^/HCV^+^ patients. We concluded that different subsets of TB-specific CD4^+^ T cells in TB/HCV-infected individuals are partially impaired in early-stage HCV infection. This was combined with increased serum IL-10 level. Such immune modulations may represent a powerful risk factor for disease progression in patients with HCV/TB coinfection.

## 1. Introduction

Infection with tuberculosis (TB) is one of the top 10 global causes of death. According to the World Health Organization (WHO) global report, TB globally infected about 9–11.1 million patients and accounted for about 1.2 million deaths among human immunodeficiency virus (HIV)-negative people in 2018. TB is endemic in Africa, which accounts for 24% of global TB cases. The incidence rate of active TB in Egyptians is 12 per 100,000 people. These numbers have been relatively stable over the last few years [1].

The hepatitis C virus (HCV) is a parentally transmitted hepatotropic virus that causes chronic infection in 55%–85% of cases. Due to its chronic nature, HCV infection usually progresses to serious end-stage complications, such as cirrhosis and hepatocellular carcinoma. Globally, about 150 million individuals are infected with HCV [2]. Moreover, Egypt is one of the countries most affected by HCV—demographic and health surveys measured antibody prevalence among the adult population to be 10% in 2015 [3].

Both TB and HCV are threatening infectious diseases and they constitute major health problems, particularly in endemic countries. The occurrence of TB/hepatitis coinfection is a significant clinical and public-health challenge, as it may cause serious health hazards and place a significant bioburden on the community. Moreover, liver damage induced by HCV increases the risk of anti-TB drug-induced hepatotoxicity. This is because liver toxicity is a common side effect of anti-TB drugs. This leads to increased complexity in the treatment of this group of TB/HCV patients [4,5]. Generally, the prevalence of HCV infection in TB patients has not been extensively analyzed. Only a very limited amount of data on the rates of TB/HCV coinfection exist [4].

Earlier studies examined the immune profiles of TB- and HCV-infected patients separately or in the presence of HIV infection. These studies showed the phenotypes and frequencies of antigen-specific CD4^+^ and CD8^+^ T-cell phenotypes in each disease [6,7]. HIV and HCV coinfections, chronic immune activation, immune dysfunction, and loss of CD4^+^ T cells favor the emergence of active TB in HIV patients. Reciprocally, the host’s immune response to TB has been shown to increase HIV-1 replication and accelerate the natural progression to AIDS [8,9,10]. However, there have been no previously published studies on the immune profiles of TB/HCV patients.

Therefore, the purpose of our study was to determine the prevalence of HCV coinfection in patients with TB in Egypt. We also aimed to explore the impact of HCV coinfection on the immune response to TB by analyzing the levels of immune activation markers IFN-γ, CD38, and HLA-DR on TB-specific CD4^+^ T cells. We also investigated whether these baseline markers could predict the response to anti-TB therapy. Since IFN-γ is predominantly expressed by the CD4^+^ subset of T cells in TB patients [11], we hypothesized that HCV coinfection would affect the host immune markers on TB-specific CD4^+^ T cells.

## 2. Materials and Methods

### 2.1. Ethics Statement

This prospective study was approved by the Regional Ethics Committee of Assiut University Hospital, Faculty of Medicine and was conducted in accordance with the provisions of the Declaration of Helsinki (approval number 054/12/2015). Informed written consent was obtained from all participants before enrolment.

### 2.2. Study Design and Setting

A total of 64 adult patients with active TB who attended the TB outpatient clinic at Assiut University Hospital from May 2017 to May 2018 were included in the study. Active TB was diagnosed by a positive microscopic examination for acid-fast bacilli and/or a positive culture on Lowenstein–Jensen media with supporting clinical findings such as chronic cough, fever, lymphadenopathy, and night sweats or radiological involvement [12].

Patients with a history of cured TB, previously treated TB, HIV coinfection, non-hepatitis C-related liver diseases, autoimmune diseases, and those who were on steroids or immunosuppressive therapy were excluded from this study. Ten sex- and age-matched healthy volunteers served as controls. They showed negative markers for hepatitis B virus (HBV) and HCV infections, and had no hepatic or chest diseases.

Recruited patients were subjected to thorough clinical assessment, abdominal ultrasonography, and laboratory tests including liver- and kidney-function tests, a complete blood count, and erythrocyte sedimentation rate (ESR). Patients were screened for HCV antibodies using an Ortho HCV Version 3.0 ELISA (Ortho Diagnostics Systems, Raritan, NJ, USA), and positive cases were confirmed and quantified using an Artus HCV-RG-RT-PCR kit (Qiagen, Hamburg, Germany).

Study subjects were subdivided into 3 groups on the basis of TB and HCV status. The first group (TB^+^HCV^−^) included patients infected with only TB, the second group (TB^+^HCV^+^) included patients with TB/HCV coinfection, and the third group (TB^−^HCV^−^) included healthy control individuals. Sera were also collected and stored at −80 °C before being assayed for IL-2, IL-4, IFN-γ, TNF-α, and IL-10 levels using ELISAs. These were performed by following the manufacturer’s instructions (R&D systems, Minneapolis, MN, USA).

All patients were followed up throughout the course of anti-TB therapy with an emphasis on their response to treatment. This was done by checking for negative tests of smear or sputum samples and clinical or radiological improvement. The anti-TB treatment regimen consisted of 2 months of 5 mg/kg isoniazid, 10 mg/kg rifampicin, 25 mg/kg pyrazinamide, and 15 mg/kg ethambutol. This was followed by 4 months of 5 mg/kg isoniazid and 10 mg/kg rifampicin.

### 2.3. Peripheral Blood Mononuclear Cell Isolation and Stimulation

Five milliliters of blood was drawn from each patient under complete aseptic conditions into heparinized tubes. Peripheral blood mononuclear cells (PBMCs) were isolated using Ficoll–Hypaque density-gradient centrifugation (Sigma-Aldrich, St. Louis, MO, USA).

PBMCs were suspended at a density of 2 × 10^6^ cells/mL in complete RPMI 1640 culture medium (Biowest, KS, USA) supplemented with 100 U/mL penicillin, 100 µg/mL streptomycin, 2 mM glutamine, and 10% heat-inactivated fetal calf serum (Gibco, MA, USA) in a 6-well plate. Cells were stimulated with TB antigens, CW and ESAT6-CFP10 peptide pools (10 μg/mL; Genemed Synthesis Inc., San Antonio, TX, USA), for 2 h. This was followed by the addition of Brefeldin A (10 μg/mL; BD Biosciences, San Jose, CA, USA) and further incubation for 16 h at 37 °C in the presence of 5% CO_2_. The solution was then analyzed by a flow cytometer.

### 2.4. Flow Cytometry and Staining

**Figure 1:**
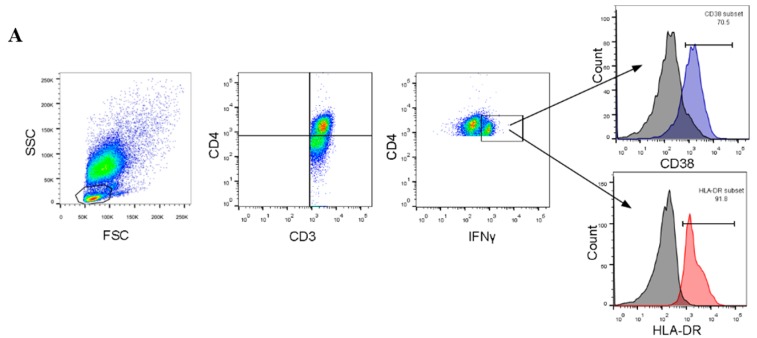

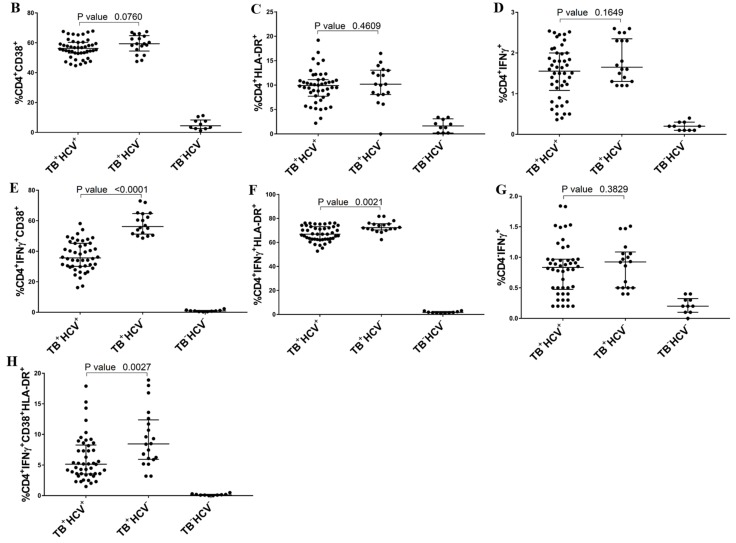
Frequencies of different T-cell subsets in different study groups. (**A**) Representative gating strategy for identifying T-cell subsets. Gray-filled histograms represent isotype controls. (**B**–**H**) Differences in frequency of different T cells expressing activation markers CD38, HLA-DR, or IFN-γ. Column bars represent median ± interquartile range; *p*-values were calculated using Mann–Whitney U-test.

After stimulation with TB proteins, PBMCs were stained with appropriate antibodies to identify different T-cell subsets specific for TB. This was done using anti-CD3 FITC (clone UCHT1), anti-CD4 PerCP/Cy5.5 (clone L200), anti-HLA-DR APC (clone 243), and anti-CD38 PE-Cy7 (clone HIT2). These were all obtained from BioLegend (San Diego, CA, USA). Cells were then fixed and permeabilized with a Cytofix/Cytoperm Kit (BD Biosciences) and stained intracellularly with anti-IFN-γ pacific blue (clone 4S.B3). Cells were acquired in a FACSCanto II (BD Biosciences), and data were analyzed using FlowJo software 7.6.1 (Tree Star Inc., Ashland, OR, USA). Figure 1A shows the gating strategy used to determine different T-cell subsets.

### 2.5. Data Collection and Statistical Analysis

All statistical analyses were carried out using SPSS for Windows version 17 (SPSS, Chicago, IL, USA). Graphs were created in Prism 5 (GraphPad, La Jolla, CA, USA). Quantitative data were expressed as mean ± standard deviation or the median and interquartile range (IQR). Data were compared using Student’s *t*-tests or Mann–Whitney U-tests for normally or abnormally distributed data, respectively. Categorical variables were expressed as a percentage and compared using a chi-squared (χ^2^) test or Fisher’s exact-probability test. Correlations were calculated using the nonparametric Spearman test. The level of significance for all statistical analyses was set at *p* < 0.05.

## 3. Results

### 3.1. Characteristics of Study Subjects

Sixty-four patients with active TB infection were included in the study. Thirty-four had active pulmonary TB, and 30 had active extrapulmonary TB. The mean age was 48.1 ± 18.9 years, and male was the predominant sex (64.1%). Out of 64 TB patients, 18 (28.1%) cases had HCV coinfection (TB^+^HCV^+^); 13 males and five females with a mean age of 54.43 ± 13.94 years. None of the HCV-infected patients had liver cirrhosis. The healthy control group (TB^−^HCV^−^) included 12 males and six females with a mean age of 44 ± 6.6 years. Regarding clinical findings, the TB^+^HCV^+^ group had a significantly lower level of serum albumin and raised level of aspartate aminotransferase (AST) compared with the TB^+^HCV^−^ group. There were otherwise no significant differences between the TB^+^HCV^+^ and TB^+^HCV^−^ groups in other clinical and laboratory parameters (Table 1). All recruited patients completed the anti-TB treatment course and a cure was obtained in 85.9% of patients (55/64). There was no significant difference between the TB^+^HCV^+^ and TB^+^HCV^−^ groups regarding their response to treatment. Baseline clinical and laboratory characteristics of the study individuals are summarized in Table 1.

### 3.2. Expression of T-Cell Activation Markers

To assess markers of T-cell activation, we calculated the percentages of different TB-specific T cells that expressed activation markers CD38, HLA-DR, and IFN-γ. The values of different analyzed T-cell subsets are shown in Figure 1B–G. The mean percentage of CD4^+^ T cells that coexpressed IFN-γ and CD38 (CD4^+^IFN-γ^+^CD38^+^ cells) was clearly lower in TB^+^HCV^+^-coinfected patients compared with TB^+^HCV^–^-mono-infected patients (36.9% ± 9.6 vs. 58.2% ± 7.6; *p*-value < 0.0001). In addition, the level of CD4^+^ T cells expressing IFN-γ and HLA-DR (CD4^+^IFN-γ^+^HLA-DR^+^ cells) was significantly lower in the TB^+^HCV^+^ group (mean = 67.2% ± 6.2) compared to the TB^+^HCV^–^ group (mean = 73.1% ± 4.7; *p* = 0.002) (Figure 1E,F). Interestingly, the frequency of CD4^+^ T cells expressing the three activation markers IFN-γ, CD38, and HLA-DR was significantly lower in the TB/HCV-coinfected group compared to the TB-monoinfected group (Figure 1H). On the other hand, analysis of the frequency of CD4^+^CD38^+^, CD4^+^HLA-DR^+^, CD4^+^IFN-γ^+^, and CD4^-^IFN-γ^+^ cells in the TB^+^HCV^+^ and TB^+^HCV^−^ groups did not reveal a statistically significant difference (Figure 1B–D and G, respectively).

Serum IL-2, IL-4, IFN-γ, TNF-α, and IL-10 levels in TB^+^HCV^+^ patients, TB^+^HCV^−^ patients, and healthy controls are shown in Table 1. The mean serum IL-10 level was significantly higher in TB^+^HCV^+^ than in TB^+^HCV^−^ patients (45.7 ± 4.1 pg/mL and 30.2 ± 2.3; *p* = 0.012). The levels of other cytokines, although higher than those in healthy controls, did not differ between TB/HCV-coinfected and TB-monoinfected patients.

### 3.3. Correlation between TB-Specific T Cells and Clinical Parameters

Analysis of the correlation between CD38, HLA-DR, and IFN-γ expression on CD4^+^ T cells and patients’ laboratory characteristics demonstrated that the level of IFN-γ^+^CD38^+^ cells was significantly correlated with the level of serum albumin (r = 0.588, *p* = 0.04). Serum bilirubin was negatively correlated with levels of IFN-γ^+^CD38^+^ (r = −0.615, *p* = 0.04) and IFN-γ^+^HLA-DR^+^ (r = −0.500, *p* = 0.05) cells. We also observed that these baseline-activation T-cell markers were not predictive for the response to antituberculous therapy among TB^+^HCV^−^ or TB^+^HCV^+^ patients—their baseline values did not show statistically significant differences between patients who responded to treatment and those who did not.

## 4. Discussion

TB and HCV are still among the most life-threatening infectious agents. They have a high mortality rate in adults particularly in developing countries where these two diseases have similar epidemiological risk factors, and sufferers share common problems regarding access to care [13,14]. Despite the importance of these pathogens, there have been few studies on HCV infection in TB patients. Most of the studies that do exist are limited to the study of hepatotoxicity accompanying anti-TB treatment [4,15,16]. In this report, a high prevalence of HCV infection (28.1%) was observed in TB patients. A previous study carried out in the Menoufiya governorate in Egypt, focusing on the impact of hepatitis on the incidence of hepatotoxicity induced by anti-TB drugs, reported an HCV/TB coinfection rate of 17% [17]. This was similar to our results.

A high prevalence of HCV (20%) has also been detected in patients with TB in four cities in Georgia. In that study, HCV coinfection was independently associated with previous incarceration and a history of receiving a tattoo [18]. In Brazil, HCV infection was seen in 7% of TB patients, while in Kurdistan, a previous study showed very low prevalence (0.9%) of HCV/TB coinfection [19,20]. Globally, a meta-analysis of published data from January 2000 to March 2018, aiming to determine the prevalence of HCV infection in patients with TB, reported that the overall prevalence of HCV infection in patients with TB was 7%. This was calculated using a random-effects model [21]. These discrepancies might be explained by the difference in geographical distribution of both diseases, and the hygienic measures of study populations. Observations regarding disease prevalence in this study were in agreement with the endemicity of these diseases in this region.

No information exists on the effect of HCV coinfection on the immune response to TB. TB/HCV coinfection presents a complex challenge to the immune system. In this study, immunological markers of TB were investigated by assessing the levels of IFN-γ^+^, CD38^+^, and HLA-DR^+^ on CD4^+^ T cells after PBMC stimulation with specific TB proteins. This was done in TB-monoinfected and TB/HCV-coinfected groups. To ensure the specificity of our findings, we also evaluated the response to cytomegalovirus (CMV). This is an unrelated antigen, and we did not observe any significant stimulation of lymphocytes (data not shown). Both CD38 and HLA-DR are markers of T-cell activation in response to bacterial infection or vaccination [22,23]. In addition, CD4^+^ T cells that express IFN-γ are central regulators in controlling TB infection and replication [24]. In our study, the total quantity of CD4^+^IFN-γ^+^ T cells was not different between TB-monoinfected and TB/HCV-coinfected groups. The main findings of this study are related to immune modulations that were observed in TB/HCV-coinfected patients. Specifically, these patients displayed decreased TB-specific IFN-γ^+^CD38^+^CD4^+^ and IFN-γ^+^HLA-DR^+^CD4^+^ T-cell levels compared to TB-monoinfected patients. This shows that the CD4^+^ T-cell immune response against TB is impaired in these individuals.

Activated CD4+ T-helper 1 cells that secrete IFN-γ and TNF-α play an important role in the defense against TB through the activation of the microbicidal functions of macrophages [25]. Petruccioli et al. [26] showed that bifunctional IFN-γ^+^TNF-α^+^ T-helper cells are significantly associated with active TB compared with latent TB. Accordingly, the lower level of IFN-γ^+^CD38^+^, IFN-γ^+^HLA-DR^+^, and IFN-γ^+^CD38^+^HLA-DR^+^ T-helper cells in TB/HCV-coinfected patients may point to a defect in the immune response against TB in this group. Interestingly, the frequency of these TB-specific IFN-γ^+^CD4^+^ T cells that express the activation markers CD38 and HLA-DR is a biomarker to distinguish active and latent TB. These cell populations are also correlated with TB burden during treatment [27].

Chen et al. [28] analyzed the effect of HBV/TB coinfection in a retrospective investigation and showed that TB/HBV patients who did not receive anti-HBV treatment were more susceptible to Grade 4 drug-induced liver injury, liver failure, and poor outcomes compared with TB-monoinfected patients. Similarly, Chukwuanukwu et al. [29] found that coinfection with TB and malaria weakened immune responses to TB and was associated with an increase in anti-inflammatory cytokine IL-10. However, coinfection may have enhanced the inflammatory response through increased T-helper 2-associated cytokines. Studies of TB/HIV-coinfected patients showed lower frequency of IFN-γ^+^ TB-specific CD4^+^ T cells in HIV-infected patients compared with TB-mono-infected individuals [30,31]. In addition, HIV infection was reported to be associated with impaired TB-specific CD4^+^ T-cell function and proliferative capacity [10]. TB is also associated with decreased HIV-specific T-cell function in TB/HIV patients [32].

Importantly, TB^+^/HCV^+^ patients had higher levels of IL-10 in this study. Previous studies showed that TB- or HCV-monoinfected patients produced IL-10 at greater levels. IL-10 could be produced by regulatory T cells as a result of infection-induced inflammation [33]. While the immunological mechanisms of persistence differ for TB and HCV, it seems that downregulation of the immune response through IL-10 secretion is a common feature in the chronic phase, which is marked by progressive immunological exhaustion [34,35]. Given the well-known role of IL-10 in the downregulation of different immune functions, such as the inhibition of interferon production and attenuation of antigen-presenting cells, higher levels of IL-10 in TB^+^/HCV^+^ patients might have pathogenic implications. This may be caused by a dampening of the T-cell response against pathogens [36,37]. Several reports showed that TB- or HCV-infected patients have higher levels of inflammatory cytokines IL-2, IL-4, IFN-γ, and TNF-α [38,39]. This was consistent with our findings.

The effect of HCV on TB-specific CD4^+^ T cells may be a mechanism for accelerated TB disease progression in TB/HCV-coinfected subjects. This is because functionally impaired CD4^+^ T cells may be unable to control TB replication, particularly when this is combined with an increased level of IL-10 in the serum.

Although we reported a difference in the frequency of TB-specific IFN-γ^+^CD38^+^ and IFN-γ^+^HLA-DR^+^ CD4^+^ T cells in the TB/HCV group, we did not observe an association between the frequency of these cells and the outcome of infection or response to anti-TB therapy. This could be explained by the moderate HCV-associated reduction in TB-specific T cells in TB^+^/HCV^+^ patients (CD4^+^IFN-γ^+^CD38^+^ and CD4^+^IFN-γ^+^HLA-DR^+^ cells were reduced by about 10%). Additionally, patients in our cohort had early-stage HCV, and none of them suffered from HCV-associated complications such as liver cirrhosis and hepatocellular carcinoma. Further studies are required to assess the effect of the immune response in patients with HCV-associated complications on the outcomes of TB infection. Moreover, only moderate levels of HCV RNA were detected (the mean level of HCV viral load in our cohort was 3.7 × 10^4^ IU/mL).

Further studies are required to elucidate the pathogenic potential of HCV/TB coinfection. This may include investigating the impact of HCV on TB latency, and whether HCV eradication can restore TB-specific T-cell levels. It is also worth analyzing the opposite situation by studying the impact of TB infection on the activity of HCV-specific T-cell responses.

## 5. Conclusions

Our data provide compelling evidence that HCV has an inhibitory effect on the immune response against TB. However, early-stage HCV infection is not associated with the development of TB-associated complications or treatment failure. Our results provide new insights into the potential mechanisms that modify the immune response in cases of TB/HCV coinfection.

## Figures and Tables

**Table 1:** Demographic, clinical, and laboratory characteristics of tuberculosis (TB) patients in this study.

Parameter	Total TB Patients (*n* = 64)	TB^+^HCV^−^ Group (*n* = 46)	TB^+^HCV^+^ Group (*n* = 18)	TB^−^HCV^−^ Controls (*n* = 18)	*p*-Value ^a^
Median age (IQR)	46.8 (21–65)	40.2 (21–63)	51.8 (28–65)	44 (25–51)	0.076
Sex M/F	41/23 (64.1%/35.9%)	28/18 (60.9%/39.61%)	13/5 (72.2%/27.8%)	12/6 (66.6%/33.3%)	0.436
Active TB Pulmonary/Extrapulmonary	35/29 (54.7%/45.3%)	25/21 (54.3/45.7%)	10/8 (55.6%/44.4%)	NA	0.296
TB toxemia	41 (64.1%)	31 (67.4%)	10 (55.6%)	NA	0.189
Fever	32 (50%)	24 (52.2%)	8 (44.4%)	NA	0.171
Weight loss	28 (43.8%)	22 (47.8%)	6 (33.3%)	NA	0.174
Serum bilirubin (mg/dL)	0.7 ± 0.3	0.8 ± 0.4	0.6 ± 0.3	0.5 ± 0.2	0.420
Serum albumin (g/dL)	3.9 ± 0.8	4.4 ± 0.5	3.5 ± 0.8	4 ± 0.5	0.03 *
AST (U/L)	31 (4–102)	22 (4–35)	43.5 (18–102)	20 (10–30)	0.004 *
ALT (U/L)	23 (10.4–98.2)	21 (10.4–87)	23 (12–98.2)	25 (15–35)	0.095
WBC (×10^9^/L)	8.1 ± 3.8	7.5 ± 3.3	8.5 ± 3.5	5 ± 0.5	0.537
Hemoglobin (g/dL)	12.9 ± 1.4	12.5 ± 1.8	13.1 ± 1.03	13.5 ± 1.53	0.288
Platelets (×10^9^/L)	307.5 ± 56.1	281.1 ± 90.7	329.5 ± 79.1	349 ± 25	0.196
Neutrophil count %	62.2 ± 8.8	58.3 ± 6.3	64.4 ± 9.6	55.3 ± 2.3	0.189
Lymphocyte count %	29.1 ± 9.4	34.1 ± 8.7	26.1 ± 8.8	30 ± 5	0.097
Serum creatinine (mg/dL)	0.9 ± 0.3	0.8 ± 0.2	1.03 ± 0.3	0.8 ± 0.2	0.342
ESR (mm/h)	52.8 ± 19.2	51.33 ± 24.56	53.71 ± 15.73	12 ± 2	0.779
HCV RNA, IU/mL	NA	NA	3.7 × 10^4^ (5 × 10^3^–8.9 × 10^6^)	NA	-
Response to anti-TB treatment	55 (85.9%)	41 (89.1%)	14 (77.8%)	NA	0.176
IL-2 (pg/mL), median (IQR)	NA	15.1 (13.2–16.6)	17.3 (17–18.4)	7.3 (6.3–8.7)	0.26
IL-4 (pg/mL), median (IQR)	NA	33 (31.5–34.2)	36 (34.3–40.3)	4.9 (4.9–6.3)	0.436
IFN-γ (pg/mL), median (IQR)	NA	50 (48–52)	45.6 (43–49)	5.3 (4.5–6.6)	0.296
TNF-α (pg/mL), median (IQR)	NA	70 (68.8–72)	64.3 (63–67.3)	9.5 (9–12)	0.189
IL-10 (pg/mL), median (IQR)	NA	45.3 (43.2–47.3)	29.3 (28.2–31.5)	4.9 (4–6.1)	0.012 *

Data expressed as mean ± standard deviation for parametric data, median (interquartile ranges) for nonparametric data, or n (%) for qualitative data. Note: HCV, hepatitis C virus; ALT, alanine aminotransferase; AST, aspartate aminotransferase; WBC, white blood cells; ESR, erythrocyte sedimentation rate; IQR, interquartile range; NA, not applicable; ^a^, *p* values were calculated between TB^+^HCV^−^ and TB^+^HCV^+^ groups; *, indicates significant difference (*p* < 0.05).
